# Combining Next-Generation Sequencing Strategies for Rapid Molecular Resource Development from an Invasive Aphid Species, *Aphis glycines*

**DOI:** 10.1371/journal.pone.0011370

**Published:** 2010-06-29

**Authors:** Xiaodong Bai, Wei Zhang, Lucia Orantes, Tae-Hwan Jun, Omprakash Mittapalli, M. A. Rouf Mian, Andrew P. Michel

**Affiliations:** 1 Department of Entomology, Ohio Agricultural Research and Development Center, The Ohio State University, Columbus, Ohio, United States of America; 2 United States Department of Agriculture - Agricultural Research Services and Department of Horticulture and Crop Sciences, The Ohio State University, Columbus, Ohio, United States of America; Temasek Life Sciences Laboratory, Singapore

## Abstract

**Background:**

Aphids are one of the most important insect taxa in terms of ecology, evolutionary biology, genetics and genomics, and interactions with endosymbionts. Additionally, many aphids are serious pest species of agricultural and horticultural plants. Recent genetic and genomic research has expanded molecular resources for many aphid species, including the whole genome sequencing of the pea aphid, *Acrythosiphon pisum*. However, the invasive soybean aphid, *Aphis glycines*, lacks in any significant molecular resources.

**Methodology/Principal Findings:**

Two next-generation sequencing technologies (Roche-454 and Illumina GA-II) were used in a combined approach to develop both transcriptomic and genomic resources, including expressed genes and molecular markers. Over 278 million bp were sequenced among the two methods, resulting in 19,293 transcripts and 56,688 genomic sequences. From this data set, 635 SNPs and 1,382 microsatellite markers were identified. For each sequencing method, different soybean aphid biotypes were used which revealed potential biotype specific markers. In addition, we uncovered 39,822 bp of sequence that were related to the obligatory endosymbiont, *Buchnera aphidicola*, as well as sequences that suggest the presence of *Hamiltonella defensa*, a facultative endosymbiont.

**Conclusions and Significance:**

Molecular resources for an invasive, non-model aphid species were generated. Additionally, the power of next-generation sequencing to uncover endosymbionts was demonstrated. The resources presented here will complement ongoing molecular studies within the Aphididae, including the pea aphid whole genome, lead to better understanding of aphid adaptation and evolution, and help provide novel targets for soybean aphid control.

## Introduction

Aphids are among the most important and intensely studied insect taxa. Species within the Aphididae represent model systems to study basic and broad biological questions including speciation and adaptation [1], [2], modification of reproduction strategies [3], and commensal linkages with bacterial symbionts [4], [5], [6], [7], [8], [9]. Additional research has focused on applied aspects of aphid biology, as some aphids are seen as important pests of agricultural and horticultural commodities [10]. Over 450 species within the family Aphididae feed on these commodities, and about 100 species are considered serious economic pests [11], [12]. Most aphids have a worldwide distribution and are highly successful invaders, benefited by asexual reproduction capability, rapid adaptability and phenotypic plasticity [1], [13], [14]. The large number of pest species within the Aphididae is no doubt aided by the aphid's complex life history and biology [11], [15].

Given the importance of the Aphididae, it is no surprise that substantial molecular resources exist for a few species [16]. Expressed sequenced tag (EST) libraries have been generated for at least 5 aphid species [17], [18], [19], [20], [21], representing nearly 160,000 total ESTs. Two species are within the largest aphid tribe, Macrosiphini: the pea aphid, *Acrythosiphon pisum* and green peach aphid, *Myzus persicae*. The remaining 3 species belong to the tribe Aphidini: the brown citrus aphid, *Toxoptera citricida*; the bird cherry-oat aphid, *Rhopalosiphum padi*; and cotton-melon aphid, *Aphis gossypii*. Gene divergence within tribes has been estimated to be 5–10%, and up to 15% when comparing among tribes [22], [23]. In addition, whole genome sequencing of the pea aphid, *Ac. pisum*, is complete [24], [25]. Annotation of the whole genome has been guided by various EST libraries, and 6,341 unique pea aphid sequences were considered orthologous when compared to the *D. melanogaster* genome [26]. Still, many pea aphid transcripts have no known function, and over 2,400 green peach aphid ESTs are completely novel and potentially aphid specific [16], [19], indicating that more aphid genomic and functional studies are necessary [24]. The release of the *Ac. pisum* genome [24] will help further advance comparative aphid research, but more representative aphid species are needed to fully understand genomic evolution in this large insect family, particularly in regards to gene duplication and molecular evolution rates [24], [27].

Although substantial genomic information existed for bacterial endosymbionts before any such aphid resources, the development of aphid genomics has led to a better understanding of the interaction between bacterial endosymbionts and host species, namely with *Buchnera aphidicola* [9], [28], [29], [30], [31]. The relationship between this primary, obligatory endosymbiont and its aphid host is thought to have occurred 100–200 mya [32], [33]. Historical as well as more recent molecular genetic studies have repeatedly shown that *Buchnera* produces essential amino acids and nutrients lacking from the insect host's diet [34], which aides in rapid adaptation to new hosts. Recent studies have also shown that gene transfer between *Buchnera* and *Ac. pisum* has been minimal, but metabolic pathway sharing is extensive [24], [31]. In addition to *Buchnera*, the presence and roles of other facultative endosymbionts such as *Hamiltonella defensa* in aphid defense and adaptation are becoming better understood [35], [36].

In this study, we present both transcriptomic and genomic resources for a less studied aphid, *Aphis glycines*. Better known as the soybean aphid, this species invaded North America in 2000 [37], presumably from its native range in Asia. Since 2000, the soybean aphid has spread across the North-Central USA and parts of Canada and has arguably become the most important insect pest of soybean in these regions. Soybean (*Glycine max* L. Merr.) is the third largest crop in USA. The estimated value of 2008 soybean crop in the USA alone was over $27 billion (USDA-NASS). Yield losses due to soybean aphid were reported to be more than 50% in Minnesota in 2001 and up to 58% in China [38], [39]. The soybean aphid life cycle in North America is similar to what is known from its native range [39]. It is a typical heteroecious, holocyclic species, alternating among sexual reproduction on primary hosts and asexual reproduction on secondary hosts [37]. On soybean, populations can double every 6–7 days [40], with close to 15 generations occurring within one growing season. Fully developed adults can produce more than 9 nymphs per day and total of more than 60 nymphs in a lifetime under laboratory conditions [41].

Despite the genetic bottleneck during the recent introduction [42], the soybean aphid has also demonstrated a remarkable ability for adaptation. Certain populations of the soybean aphid have adapted to overcome resistant genes in newly developed soybean lines [43], [44], [45], [46]. Termed biotypes, at least two types have been described: Biotype 1 (IL) and Biotype 2 (OH) [47]. Biotype 1 does not survive and reproduce on soybeans with the *Rag*1 resistance gene whereas Biotype 2 does. Although the formation of biotypes is fairly common within Aphididae—14 species have at least 2 described biotypes each [10] —little is known regarding the molecular mechanisms of biotype adaptation to resistant crop plants in any aphid species. Generating molecular resources for the soybean aphid would not only complement the array of aphid resources already available, but, if genes responsible for biotype adaptation could be identified, it could streamline efforts in developing resistant soybean and sustain the durability of these varieties, thereby helping control one of the most important pests of soybean in North America using genomic tools.

The current next-generation sequencing technologies offer a prime opportunity to generate molecular resources for many non-model species [48], [49]. These methods vary in their applicability in terms of research questions and molecular resources required [48], [50]. The Illumina technology provides high coverage but short reads and is most efficient for species that have substantial sequence information to serve as a reference and aid in assembly. Data generated by Roche-454 sequencing produces longer fragments and may be more suited for species without significant sequence data already available [49], but overall sequencing output and coverage is lower than Illumina [48]. Prior to our study, sequence information for the soybean aphid was limited. Therefore, due to difficulties of assembly without a reference sequence, using an Illumina approach may result in a low number of long fragments and be less informative than 454. However, because of the genetic bottleneck during the North American invasion, the low overall output and coverage with 454 as compared to Illumina may result in a smaller number of sequences for the generation of high-quality molecular markers, *e.g.* microsatellites, single nucleotide polymorphism (SNPs), for population-level genetic analyses. These issues become readily apparent for non-model invasive species, whose importance is heightened due to the environmental and ecological impacts on the invaded regions. For the soybean aphid in particular, maximizing outputs from sequencing projects is difficult because of a genetic bottleneck and a lack of molecular resources.

Our goal was to rapidly generate molecular resources for the soybean aphid and find potential candidate genes responsible for biotype adaptation. In this study, we used multiple platforms: Illumina technology to target genomic, non-coding, and potentially more polymorphic regions and Roche-454 technology to generate longer reads from the transcriptome, which would allow a more robust characterization to other aphid EST libraries. In addition, we used a different biotype as starting material for each technology that uncovered potential diagnostic markers related to biotype adaptation.

## Methods

### *A. glycines* culture maintenance

The two soybean aphid biotypes used for this study, Biotype 1 (IL) and Biotype 2 (OH), show differential responses to soybean plants containing the soybean aphid resistance gene, *Rag*1 [47]. Biotype 1 (IL), hereafter B1, cannot survive or reproduce on *Rag*1, whereas Biotype 2 (OH), hereafter B2, can survive and reproduce on *Rag*1. These biotypes are housed in 2 different locations at The Ohio Agricultural Research and Development Center (OARDC) to prevent contamination. B1 individuals were obtained from the National Soybean Research Laboratory, Department of Crop Sciences, University of Illinois, Urbana-Champaign in February 2008. An OARDC laboratory population was established from these initial individuals. The laboratory population of B2 was established from field collected aphids from Wooster, OH in 2005. The aphid laboratory populations were maintained on cultivar Williams 82 (susceptible to both biotypes) in growth chambers or rearing rooms at temperatures between 22 and 24°C, with a photosynthetically active radiation of 330 µmol m^−2^s^−1^ for 15 h daily and 60 to 70% relative humidity [51]. Laboratory populations are routinely checked on resistant whole plants to ensure responses to resistant soybean plants have not changed (every 6 months). Individuals from each laboratory populations used in the current study were randomly collected from multiple soybean plants.

### *A. glycines* B1 454 cDNA library construction and sequencing

Only B1 aphids were used for the 454 transcript sequencing. Total RNA was isolated from 50 aphids with Trizol Reagent (Invitrogen). Approximately 10–20 µg total RNA were sent to Purdue University Genome Center for 454 cDNA library construction and sequencing using ¼ of a pico titer plate. Briefly, the poly(A) RNA was collected using RiboMinus (Invitrogen) kit following manufacturer's instructions. Double-stranded cDNA was synthesized using cDNA Synthesis System Kit (Roche) and separated in an agarose gel. The DNA bands of 500–800 bp were excised from the gel and purified. The isolated DNA was blunt ended, ligated to adapters and immobilized on Library Immobilization Beads. After the gaps were repaired, a single-stranded DNA library was isolated from the beads and quality controlled for the correct size using a LabChip 7500 machine. The concentration and the proper ligation of the adapters were examined using qPCR. The library was subjected to sequencing using Roche 454 GS Titanium platform using 1/4according to manufacturer's protocol.

### Reduced representation genomic library of *A. glycines* B2

Only B2 aphids were used for Illumina sequencing. DNA was extracted from 25–30 aphids of the B2 laboratory population using the OMEGA EZNA DNA tissue kit (Doraville, GA) following manufacturer's instructions. Whole extractions were then subjected to restriction digests with 5 µg of DNA and 25 U of *Eco*RI in a total of 25 µL. Restriction digests were run overnight to ensure complete digestions. DNA was then electrophoresed in a 1% agarose gel, and the fragments 2–3 Kb in size were extracted and purified using the QIAquick Gel Extraction Kit (Qiagen). The procedure was repeated with 3 separate reactions to obtain a total of 1 µg DNA. DNA was then fragmented into 200 bp using the Adaptive Focused Acoustics technology from Covaris ™ (Woburn, MA) and then purified using QIAquick PCR Purification Kit (Qiagen). Using only the 2–3 Kb fragments resulted in a “reduced representation” library [52]. Although only a small portion of the genome was sequenced, the advantage of such reduced representation is an increase of coverage per contig.

About 1 µg of the 200-bp fragments was used to prepare Illumina paired-end library using Paired-End Sequencing Sample Preparation Kit (Illumina) following manufacturer's instructions. Briefly, the DNA fragments were end-repaired and ‘A’ bases were added to the 3′-end of the DNA fragments, followed by the ligation of Illumina adapters. After purification, a 10 cycle enrichment of the adapter-modified fragments was performed. The library was validated by measuring 260/280 ratio using a Nanodrop D-1000 spectrophotometer and TBE polyacrylamide gel electrophoresis. The validated library was sent for sequencing using a single lane on an Illumina GAII.

### Bioinformatic data analysis

The 454 transcript data from the *A. glycines* B1 were assembled using Newbler program (Roche) after the removal of adapter sequences. The contigs and singletons were further assembled using Phrap program. To achieve better consistency, the contigs and singletons were renamed in the format of “ESTAGB1WB000001” with “EST” standing for expressed sequence tag, “AG” for *Aphis glycines*, “B1” for B1, “WB” for whole body library, and “000001” for an arbitrarily assigned number.

The 51-bp Illumina paired-end data of the reduced representation genomic library of *A. glycines* B2 were assembled using velvet program [53]. The paired-end sequences have both the sequence and the position information, which ensure better *de novo* assembly than single-end sequences (Illumina, Inc.). We selected the contigs with a length cutoff of 500 bp for further analysis to limit the inclusion of contaminant or low quality sequences and to ensure adequate flanking sequence was present to design PCR primers for microsatellite and SNP analysis. To achieve better consistency, these contigs were renamed in the format of “GMAGB2RR000001” with “GM” standing for genomic, “AG” for *Aphis glycines*, “B2” for B2, “RR” for reduced representation library, and “000001” for an arbitrarily assigned number.

The transcriptome sequences of *A. glycines* B1 and the genomic sequences of *A. glycines* B2 were annotated by searching against GenBank non-redundant database using BLASTx algorithms [54]. The *A. glycines* B1 transcriptome sequences were searched against the draft genome (Acyr_1.0) of pea aphid *Ac. pisum* (http://www.aphidbase.com/aphidbase) using BLASTn algorithm [54]. The domains in the sequence were identified by searching against Pfam database release 24.0 [55] using HMMER v3 program [56] with an E value cutoff of 1e-5.

### Molecular Marker Detection and Analysis

The SNPs in *A. glycines* B2 Illumina data were predicted by MAQ program (http://maq.sourceforge.net/) with the default 3-read threshold for SNP calling. The SNPs in *A. glycines* B1 454 data were predicted by gsMapper program (Roche) with an arbitrary criterion of at least 4 reads supporting the consensus or variant. We also identified the SNPs between the two biotypes by mapping the 454 raw reads to the Illumina contigs using the gsMapper program with the above settings.

The Allele Specific Primer Extension (ASPE) assay was used for validation of 11 SNPs. Briefly, the ASPE entails 2 rounds of PCR: one to amplify sequence flanking the SNP, and a second, ASPE- PCR. For the ASPE-PCR reaction, the allele-specific primers are tagged, allowing linking of fluorescently labeled polystyrene microspheres, with each SNP allele associated with a different color [57], [58]. All primers were designed using Primer 3 [59]. The initial PCR was performed with 2× master-mix buffer, 5 pmol of forward (F) and reverse (R) primers, and 1.0 uL of DNA (∼10 ng) in 15 µL reactions. Cycling conditions were an initial 94° for 2 min, then 30 cycles of 94°C/30 s; 58°C/30 s; 72°C/60 s, followed by 72° for 10 minutes. The ASPE amplifications were multi-plexed using 500 nmols of primer for each of the 11 loci, 10× ASPE buffer, 0.075 µL of Tsp DNA polymerase, 0.5 µL dNTP 20× mix, 0.125 µL of 400 uM biotin-dCTP, and pooled PCR products as templates. The median fluorescent intensity (MFI) emitted by the Luminex analysis was used to determine the presence or absence of each SNP in the amplified sample. Data was normalized by dividing the MFI of each allele by the sum of the MFI of both alleles [60]. A total of 48 total individuals were tested, 8 each from 6 populations.

The microsatellite markers were identified using the msatfinder program with the minimal repeat number of 8 for di-nucleotide motifs and 5 for motifs consisting of three and more nucleotides [61]. The Blast2GO program [62], [63] was used to predict the functions of the sequences, assign Gene Ontology terms, and predict the metabolic pathways in KEGG [64], [65], [66].

### Endosymbiont PCR confirmation

For *Hamiltonella defensa* sequence confirmation, primers were designed using Primer 3 [59], and with the PCR conditions of 94°C for 5′, followed by 30 cycles of 94°/30 s, 55/15 s, and 72°/1′. Reactions were performed in 20 µL total volume with 10 µL of 10× reaction buffer (Failsafe polymerase chain reaction [PCR] premix; Epicenter Technologies, Madison, WI), 4 pmol of each primer, 1 U of Taq Polymerase (Genscript, Piscataway, NJ), and 1 µL of soybean aphid DNA template (8 ng/µL). A total of 16 soybean aphid individuals were tested; 8 from each biotype. For 11 individuals (4 of B1 and 7 of B2), PCR reactions were purified using agarose gel extraction kits (Qiagen), and sent for direct sequencing using an AB1 3730XL (Functional Biosciences, Inc., Madison, WI).

### Data Deposition

The raw 454 transcript reads of *A. glycines* B1 and raw Illumina genomic reads of *A. glycines* B2 were deposited in NCBI Sequence Read Archive under the accession number of SRA010354.

## Results and Discussion

### Sequencing Analysis and Gene Characterization

**Figure 1:**
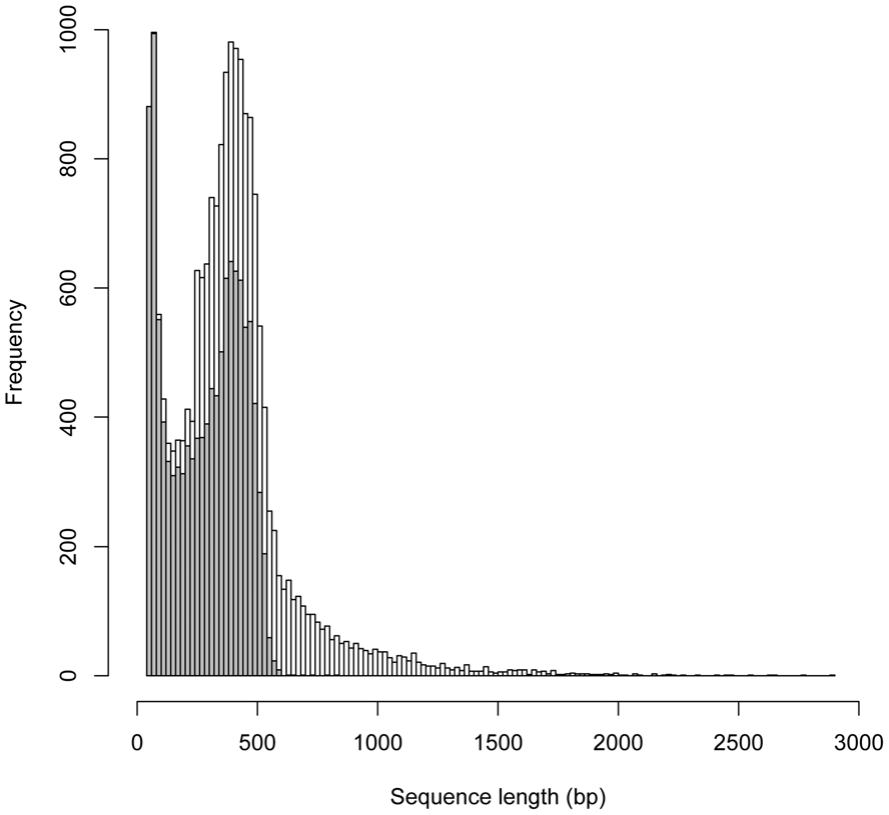
Length distribution of *Aphis glycines* B1 transcripts from 454 sequencing. The length distribution of soybean aphid *Aphis glycines* transcript sequences. The grey bars represent the singleton sequences and the white bars represent contig sequences.

The 454 sequencing for *A. glycines* B1 yielded 102,024 transcript reads totaling 30,438,043 bp. After the removal of adapter sequences, the trimmed sequences went through two rounds of assembly using Newbler and phrap programs, resulting in 19,293 high quality transcript sequences totaling 7,366,599 bp. In total, the 454 transcript data consisted of 7,427 contigs (>1 read after assembly) and 11,866 singletons (a single read after assembly). The singletons ranged from 50–824 bp with an average length of 281 bp and total length of 3,334,913 bp (Figure 1). The contigs ranged from 61–2,893 bp with an average length of 542 bp and total length of 4,031,686 bp.

**Figure 2:**
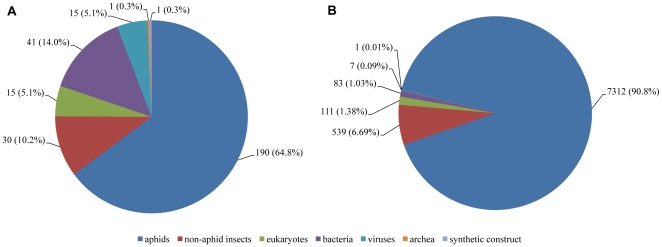
Species distribution of top BLASTx matches of *Aphis glycines* sequences. Species distribution of the top BLASTx matches of the transcripts of *A. glycines* B1 (A) and reduced representation genome of *A. glycines* B2 (B). The percentages were calculated considering the total number of sequences with BLASTx hits as 100%.

Among the 19,293 high quality transcript contigs and singletons, of *A. glycines* B1, 8,053 (42%) matched to proteins in the GenBank nr database with the E value cutoff of 1e-5 (Figure 2A). A vast majority (7,310; 91%) of the top matches were to proteins of aphids, mainly pea aphid *Ac. pisum*. Another 539 matches (7%) were to non-aphid insect proteins. The rest of the matches were to proteins of non-insect eukaryotes, bacteria, viruses, and synthetic construct. At the nucleotide level, 13,818 transcripts (72%) matched to the *Ac. pisum* draft genome based on the BLASTn search with an E value cutoff of 1e-5. A comparison with the ESTs of *A. gossypii* using tBLASTx algorithm revealed 3,875 (20%) of *A. glycines* B1 transcripts had significant similarity (E value cutoff of 1e-5) to *A. gossypii* sequences.

The reduced representation genomic sequencing for *A. glycines* B2 yielded 2,437,477 paired-end reads that were 51 bp in length, totaling 248,622,654 bp. The assembly using velvet program resulted in 56,688 contigs of 8,994,108 bp, ranging from 61 to 2,680 bp in contig length. Only the 1,240 contigs of over 500 bp in length, totaling 881,864 bp, were selected for the molecular marker prediction to ensure adequate flanking sequence for PCR primer design. While both transcripts and genomic sequences were used for molecular marker predictions, gene annotation of the genomic sequences was not performed because the intron-containing genomic sequences were too short for informative gene prediction and annotation.

Among the 1,240 contigs of *A. glycines* B2, 293 (24%) had significant matches (E value threshold of 1e-5) to proteins in GenBank nr database (Figure 2B). A significant portion (65%) of the matches was to proteins of aphids. Other matches were to non-aphid insects (11%), non-insect eukaryotes (5%), bacteria (14%), and viruses (5%). There was also one sequence matching to an *Archea* protein and one to synthetic construct. The BLASTn search against *Ac. pisum* genomic sequences revealed that 1,003 (81%) *A. glycines* B2 contigs had sequence similarity (E value cutoff of 1e-5) to *Ac. pisum* genomic sequences.

The genome size of the soybean aphid is unknown, although estimate from other *Aphis* spp. range from 479 Mbp to 655 Mbp [67], [68]. Therefore, coverage estimates are currently difficult to calculate. However, these results showed significant similarity between *A. glycines* and *Ac. pisum* at the nucleotide and protein (deduced amino acid) levels. Despite this similarity, 28% of *A. glycines* B1 sequences and 19% of *A. glycines* B2 sequences had no similar sequences with the current *Ac. pisum* draft genome. Of the 28% (5,475) of *A. glycines* B1 transcriptomic sequences, 75 matched to *Ac. pisum* proteins, 154 to proteins of other organisms, and the remaining 5,246 had no significant similarity to any proteins in GenBank nr database. Among the 19% (237) of *A. glycines* B2 genomic sequences that do not have similar sequences in the current *Ac. pisum* draft genome, 16 matched to *Ac. pisum* proteins, 59 to proteins of other organisms, and 162 had no significant similarity to any proteins in the GenBank nr database. The sequences with potential bacterial, viral, and artificial origins were removed from further annotation and molecular marker development.

### Gene Ontologies, Pathways and Protein Domains

Gene Ontology (GO) terms were assigned to 3,031 transcripts of *A. glycines* B1 (Table S1). The GO assignment included 1,760 biological process terms assigned 6,543 times to 2,176 *A. glycines* transcripts, 450 cellular component terms assigned 3,468 times to 1,973 transcripts, and 812 molecular function terms assigned 5,196 times to 2,571 transcripts. “Oxidation reduction” (102) was the most dominant biological process term, while the most dominant molecular function and cellular component terms were “protein binding” and “cytoplasma”, respectively. The GO terms were summarized according to the top-level terms (Figure S1). The top-level terms of “cellular process”, “metabolic process”, “biological regulation”, and “developmental process” were the most abundant ones in biological process category. About 50% of the transcripts being assigned with molecular function terms were involved in binding activity and 34% involved in catalytic activity. A plausible explanation for the high level of transcripts involved in oxidation-reduction processes could be attributed toward the presence of oxidative stress. The soybean aphid may encounter a high level of reactive oxygen species (ROS) during feeding on the host plant (exogenous source) as well as deal with endogenous sources of ROS such as respiration [69], [70]. The presence of transcripts encoding a suite of antioxidant response enzymes such as superoxide dismutase, glutathione peroxidases, and cytochrome P450, within the 454 transcript data further support this hypothesis.

There were 564 unique components of 112 KEGG metabolic pathways in the *A. glycines* B1 transcripts (Table S2). A large number of these sequences were products involved in important processes for nucleotide biosynthesis and metabolism such as purine (101 transcripts encoding 36 enzymes) or pyrimidine biosynthesis (61 transcripts encoding 20 enzymes).

A total of 2,024 putative domains were identified in the translated sequences of 3,987 transcripts by searching against the Pfam database release 24.0 (Table S3). The most abundant domain was the WD40 repeat, which represents a large family of eukaryotic proteins that are involved in a variety of functions ranging from signal transduction and transcription regulation to cell cycle control and apoptosis [71]. WD40-repeat protein is also involved in protein-protein interactions. Other highly abundant domains included RNA recognition motif, protein kinase domains, insect cuticle protein domain, protein tyrosine kinase domain, Ras family domain, and C2H2-type zinc finger domain.

The piwi domain was identified in 4 transcripts and the PAZ domain in 1 transcript. These two domains are typically present in the components of RNA induced silencing complex (RISC), which digest single-stranded RNA based on sequence similarity to the microRNA or siRNA involved in RNA interference (RNAi) [72], [73]. Although there is limited success of RNAi in aphids [24], [74], the identification of these domains in the soybean aphid could lead to the development of this reverse genetics tool for learning gene function and responses to aphid resistant soybeans.

### Molecular Marker Development

A total of 430 putative SNPs in 239 contigs were identified in the reduced represented genomic sequences of *A. glycines* B2 (Table S4) and 155 Putative SNPs in *A. glycines* B1 transcripts (Table S5). The types of the SNPs and allele frequencies are summarized in Table 1. A total of 11 SNPs were analyzed in 48 field-caught individuals of the soybean aphid. For each SNP tested, we verified the SNP at the position that was predicted based on our *in silico* analyses. Table 2 reports allele frequencies for each SNP, with the least common allele ranging from 0.19 to 0.47 in overall frequency. Even within this small sample (48 individuals), 9 out of 11 SNPs were polymorphic.

**Table 1:** Type of putative SNPs identified in soybean aphid *A. glycines*.

	B1	B2	B1 and B2
Transition			
A-G	67	162	17
C-T	46	148	13
Transversion			
A-C	4	35	8
A-T	25	58	3
C-G	2	14	5
G-T	11	26	4
Total	155	443	50

**Table 2:** SNP allele frequencies.

	SNP	Population					
Locus	Allele	SD	MN	ON	IA	OH	MI
SNP 6248	G	0.50	0.25	0.25	0.31	0.69	0.44
	T	0.50	0.75	0.75	0.69	0.31	0.56
SNP 3383	C	0.63	0.44	0.44	0.88	0.38	0.44
	T	0.38	0.56	0.56	0.13	0.63	0.56
SNP 5820	C	0.63	0.44	0.50	0.50	0.38	0.50
	T	0.38	0.56	0.50	0.50	0.63	0.50
SNP 42701	A	0.31	0.44	0.25	0.38	0.38	0.25
	T	0.69	0.56	0.75	0.63	0.63	0.75
SNP 7006	A	0.50	0.44	1.00	0.56	0.56	0.75
	T	0.50	0.56	0.00	0.44	0.44	0.25
SNP 2816	A	0.19	0.19	0.31	0.06	0.13	0.38
	G	0.81	0.81	0.69	0.94	0.88	0.63
SNP 8815	A	0.31	0.25	0.38	0.44	0.63	0.50
	G	0.69	0.75	0.63	0.56	0.38	0.50
SNP 2836	A	0.19	0.50	0.50	0.44	0.56	0.50
	T	0.81	0.50	0.50	0.56	0.44	0.50
SNP 47077	A	0.31	0.00	0.06	0.25	0.06	0.44
	G	0.69	1.00	0.94	0.75	0.94	0.56

SD: South Dakota; MN: Minnesota; ON: Ontario; IA: Iowa; OH: Ohio; MI: Michigan.

All populations contained 8 individuals.

We also identified 50 putative SNPs between *A. glycines* B1 and B2 (Table S6). These 50 SNPs were distributed among 25 sequences. BLASTx revealed that 8 of these sequences had no known match in NCBI, despite coming from *A. glycines* transcripts. The remaining 13 sequences were related to *Ac. pisum*, but did not significantly match to any known proteins. The presence of these sequences in the transcript data suggests that they may be unique to *A. glycines*. Further research is ongoing to compare these SNPs to field-caught soybean aphids and determine if any can be used as molecular diagnostic for biotypes as well as their possible involvement in response to soybean feeding.

For microsatellites, 1,024 loci were detected in the transcriptomic sequences of *A. glycines* B1 and 358 microsatellite loci in the reduced represented genomic sequences of *A. glycines* B2. The vast majority of the microsatellite loci (96% for B1 and 99% for B2) were di- or tri-nucleotide repeats (Table 3). The properties of the microsatellite loci including the start, stop, motif units, footprint, repeat units, motif type, and whether primers can be designed are summarized in Table S7. Full details of designing and testing of microsatellite primers from these sequences are in progress and will be published elsewhere. Preliminary results indicate at least 120 (94%) of 128 primers designed from the *A. glycines* B2 sequences amplified PCR products of expected sizes from *A. glycines* DNA samples. The large amount of potential molecular markers found in this study will enable more detailed population genomic studies of *A. glycines* to track and isolate regions of adaptive divergence [75].

**Table 3:** Summary of microsatellite loci predicted in *A. glycines* sequences.

Number of repeats	Di-nucleotide repeats		Tri-nucleotide repeats		Tetra-nucleotide repeats		Penta-nucleotide repeats	
	B1	B2	B1	B2	B1	B2	B1	B2
5			343	107	23	1	5	1*
6			165	41	9	1	1	
7			118	29				
8	85	67	61	19	1			
9	49	24	32	9				
10	27	23	21	1				
11	19	17	13					
12	8	15	10		1			
13	11	13	5					
14	3	2	3					
15	4		1					
16			2					
17	1		1					
18	1		1					
…								
21			1					

*This locus is a hex-nucleotide repeat.

### Endosymbiont sequences

We identified 83 sequences among the *A. glycines* B1 transcripts and 41 sequences in the reduced represented genome of *A. glycines* B2 that represented putative prokaryotic endosymbiont sequences. Among them, 48 transcripts from *A. glycines* B1 and 21 sequences from *A. glycines* B2 matched to proteins of *B. aphidicola*, the well-known endosymbiont of aphids [6], [7] (Table S8). One sequence, *groEL*, was shared and identical among biotypes, resulting in a total of 68 *Buchnera* genes (39,822 total bp, 6.2% of the genome, based on 640,681 bp genome [9]). *Buchnera* is known to play roles in adaptation and linked to biotype formation in other aphid species [7], [30], [76]. For example, *Buchnera* genes previously implicated in adaptation to heat stress (*groEL, dnaK,*) were found in the *Schizaphis graminum* [77]. In addition, we uncovered genes that are involved in amino acid synthesis and nutrient supplementation (*trpG, trpE, trpD, argS, dapF, ilvC, leuS, aroE, hisG*, [78]). Given that *Buchnera* is responsible for the synthesis of essential amino acids in the aphid's diet, these genes could play a role in the different biotype responses on resistant soybeans.

We also detected a fragment (1.2 kb) with similarity to *Hamiltonella defensa*. PCR amplification of this fragment was successful in all 16 additional soybean aphid individuals tested. DNA sequencing revealed exact matches to the previous sequences, and no differences among biotypes. *H. defensa* is a facultative symbiont of other phloem-feeding insects and its 2.1 Mbp genome showed dramatic genome reduction compared to its close free-living relatives of *Yersinia* and *Serratia* species [79]. A previous study tested for the presence of 3 facultative symbionts in *A. glycines (Serratia symbiotica*, *Regiella insecticola* and *H. defensa*) using universal primers spanning the intergenic spacer between the 16S and 23S rDNA genes [80]. Consistent with the present study, neither *S. symbiotica* nor *R. insecticola* were found. While a *H. defensa* PCR fragment was amplified using conserved rDNA primers, sequencing of this fragment revealed more similarity to *Arsenophonus*, another facultative endosymbiont not found in either our 454 or reduced representation libraries. Given the close genetic similarity between *Hamiltonella* and *Arsenophonus*, it is possible that preferential amplification occurred with the universal rDNA primers, decreasing the chance of *H. defensa* detection through standard PCR [80]. In addition, the different populations used between studies may harbor different populations of facultative endosymbionts, as some endosymbionts are either lost or obtained based on environmental conditions and horizontal transmission [81]. Regardless, next generation sequencing, as well as subsequent PCR and re-sequencing, confirmed that *A. glycines* harbors *H. defensa*.

### Conclusion

A major advantage of next generation sequencing technologies is the rapid and inexpensive generation of molecular resources relative to traditional methods. Choosing among technologies to maximize information content depends not only on the research interests and needs but on the organism's biology as well. In the case of the soybean aphid, the recent bottleneck during the North American invasion severely decreased genetic diversity [42], and no significant prior sequences were available. In addition, soybean aphid populations were adapting, despite the lack of genetic diversity. Thus, by combining both long and short read sequencing technologies and using different biotypes as starting material, we were able to generate significant molecular resources for the soybean aphid, as well as detect the presence of bacterial endosymbionts. This approach reveled hundreds of molecular markers for population-level analyses, potential diagnostic differences among biotypes, candidate genes involved in host-plant resistance, and a wide-array of endosymbiont sequences. The molecular resources presented in this study will provide many novel targets for soybean aphid control, as well as contribute to the expanding knowledge of the biology, ecology and evolution of the Aphididae, one of the most important insect taxa.

## Supporting Information

Figure S1 Summary of the top-level Gene Ontology terms of *Aphis glycines* B1 transcript sequences. Summary of top-level GO terms of (A) Biological Process, (B) Cellular Component, and (C) Molecular Function assigned to *A. glycines* B1 transcript sequences. Percentage was calculated by considering the total number of term assignment in that category, which is larger than the number of transcripts assigned terms in that category, as 100%.
(2.84 MB TIF)

Table S1 Gene Ontology assignment for *Aphis glycines* B1 transcript sequences
(0.61 MB XLS)

Table S2 Metabolic pathways for *Aphis glycines* B1 transcript sequences in Kyoto Encyclopedia of Genes and Genome (KEGG)
(0.06 MB XLS)

Table S3 Predicted Pfam domains in *Aphis glycines* B1 transcript sequences
(0.64 MB XLS)

Table S4 Single nucleotide polymorphisms (SNPs) predicted in *Aphis glycines* B2 genomic sequences
(0.07 MB XLS)

Table S5 Single nucleotide polymorphisms (SNPs) predicted in *Aphis glycines* B1 transcript sequences
(0.03 MB XLS)

Table S6 Single nucleotide polymorphisms (SNPs) predicted between *Aphis glycines* B1 and B2 sequences
(0.02 MB XLS)

Table S7 Microsatellite loci predicted in *Aphis glycines* B1 and B2 sequences
(0.21 MB XLS)

Table S8 Potential Buchnera sequences in *Aphis glycines* B1 and B2 sequences
(0.03 MB XLS)
