# Supplementary figures and images for: Combining Next-Generation Sequencing Strategies for Rapid Molecular Resource Development from an Invasive Aphid Species, Aphis glycines
Source: PLoS One. 2010 Jun 29;5(6):e11370. doi: 10.1371/journal.pone.0011370 (PMC2894077; doi:10.1371/journal.pone.0011370)

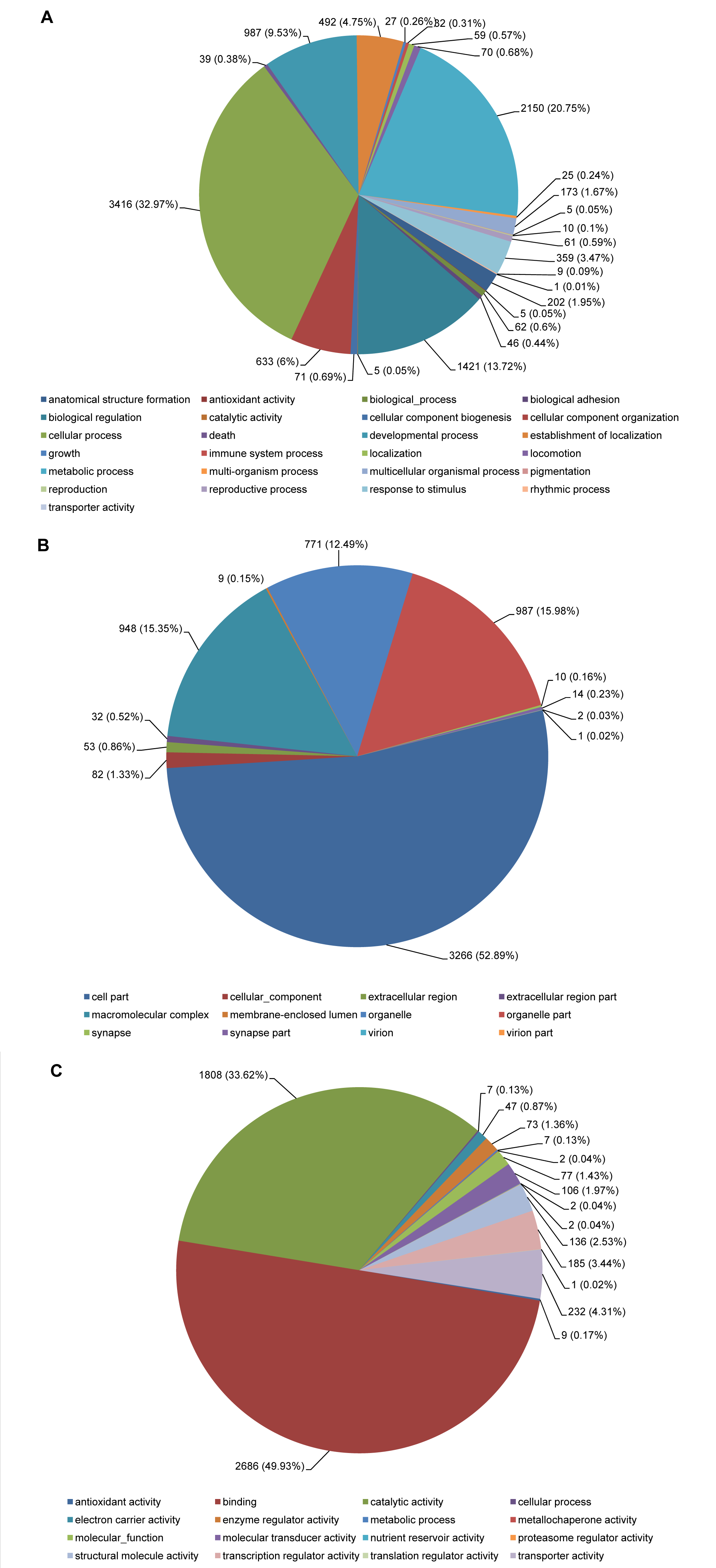

Supplement: Figure S1 — Summary of the top-level Gene Ontology terms of Aphis glycines B1 transcript sequences. Summary of top-level GO terms of (A) Biological Process, (B) Cellular Component, and (C) Molecular Function assigned to A. glycines B1 transcript sequences. Percentage was calculated by considering the total number of term assignment in that category, which is larger than the number of transcripts assigned terms in that category, as 100%. (2.84 MB TIF) [file pone.0011370.s001.tif]
